# Medicare's hospital readmissions reduction program and the rise in observation stays

**DOI:** 10.1111/1475-6773.14142

**Published:** 2023-02-27

**Authors:** Brad Wright, Canada Parrish, Anirban Basu, Karen E. Joynt Maddox, Joshua M. Liao, Amber K. Sabbatini

**Affiliations:** ^1^ Department of Health Services Policy and Management Arnold School of Public Health, University of South Carolina Columbia South Carolina USA; ^2^ Department of Health Systems and Population Health University of Washington School of Public Health and Department of Emergency Medicine, University of Washington School of Medicine Seattle Washington USA; ^3^ Department of Pharmacy and The Comparative Health Outcomes, Policy, and Economics (CHOICE) Institute University of Washington School of Pharmacy Seattle Washington USA; ^4^ Department of Medicine Washington University School of Medicine in St. Louis and Center for Health Economics and Policy, Institute for Public Health, Washington University in St. Louis St. Louis Missouri USA; ^5^ Department of Health Systems and Population Health University of Washington School of Public Health Seattle Washington USA; ^6^ Department of Medicine University of Washington School of Medicine Seattle Washington USA; ^7^ Department of Emergency Medicine University of Washington School of Medicine Seattle Washington USA

**Keywords:** difference‐in‐differences, hospital readmissions reduction program, inpatient, Medicare, observation stays, recovery audit contractors

## Abstract

**Objective:**

To evaluate whether Medicare's Hospital Readmissions Reduction Program (HRRP) is associated with increased observation stay use.

**Data Sources and Study Setting:**

A nationally representative sample of fee‐for‐service Medicare claims, January 2009–September 2016.

**Study Design:**

Using a difference‐in‐difference (DID) design, we modeled changes in observation stays as a proportion of total hospitalizations, separately comparing the initial (acute myocardial infarction, pneumonia, heart failure) and subsequent (chronic obstructive pulmonary disease) target conditions with a control group of nontarget conditions. Each model used 3 time periods: baseline (15 months before program announcement), an intervening period between announcement and implementation, and a 2‐year post‐implementation period, with specific dates defined by HRRP policies.

**Data Collection/Extraction Methods:**

We derived a 20% random sample of all hospitalizations for beneficiaries continuously enrolled for 12 months before hospitalization (*N* = 7,162,189).

**Principal Findings:**

Observation stays increased similarly for the initial HRRP target and nontarget conditions in the intervening period (0.01% points per month [95% CI −0.01, 0.3]). Post‐implementation, observation stays increased significantly more for target versus nontarget conditions, but the difference is quite small (0.02% points per month [95% CI 0.002, 0.04]). Results for the COPD analysis were statistically insignificant in both policy periods.

**Conclusions:**

The increase in observation stays is likely due to other factors, including audit activity and clinical advances.

**What is known on this topic**

- Observation stays have increased significantly over the last 15 years.
- Observation stays are excluded from the numerator and denominator of readmissions measures.
- Medicare's Hospital Readmissions Reduction Program (HRRP) may incentivize hospitals to substitute observation stays for inpatient readmissions, although evidence of this is mixed.

**What this study adds**

- Ours is the first study to examine how hospitals may have responded to the HRRP by using observation stays in lieu of both index admissions and readmissions.
- Using quasi‐experimental methods, we find that the HRRP was not meaningfully associated with differential increases in the use of observation stays for target versus nontarget conditions.
- The increase in observation stays is more likely the result of advances in clinical care and hospitals taking steps to avoid payment audits.

## 1 INTRODUCTION

Medicare's Hospital Readmissions Reduction Program (HRRP) has been controversial since its announcement. One ongoing concern is whether the HRRP incentivizes hospitals to use observation stays to avoid readmission penalties, as observation stays are excluded from the numerator and denominator of readmission measures.^1^ Approximately 23% of 30‐day return hospitalizations—and 18% of those with HRRP target conditions—are not captured in readmissions performance because they occurred after discharge from an observation stay or a patient's return hospitalization was classified as observation.^2^

Only some HRRP studies have found 30‐day readmission and observation rates to be negatively correlated.^3^, ^4^, ^5^ One regression discontinuity evaluation found nonpenalized hospitals used significantly more observation stays for heart failure patients just before versus after the 30‐day HRRP cutoff.^6^ Observation stays at nonpenalized hospitals were longer, suggesting potential substitution of observation stays for otherwise longer or more complex inpatient readmissions.^7^ Importantly, all prior evaluations only considered observation stays in the 30 days following discharge from an inpatient admission. To date, no studies have examined how hospitals may have responded to the HRRP by using observation stays in lieu of both index admissions and readmissions.

In this study, we used a difference‐in‐difference (DID) approach to examine whether HRRP implementation was associated with an increased rate of observation stay use among Medicare beneficiaries.

## 2 METHODS

Using a 20% sample of fee‐for‐service Medicare claims between January 1, 2009 and December 31, 2016, we identified inpatient admissions and hospital observation stays to short‐term general and specialty hospitals for beneficiaries continuously enrolled for 12 months prior to hospitalization. Although these data are more than 5 years old, we use them because they include the periods immediately before and after HRRP announcement and implementation. We derived our sample by adapting the inclusion and exclusion criteria of CMS' hospital‐wide readmission measure.^8^ We excluded all hospitalizations to cancer specialty hospitals, critical access hospitals, and Maryland hospitals (both of which are exempt from the HRRP), and hospitals with fewer than 100 inpatient admissions in the prior year (to avoid unstable estimates). We also excluded 96 outlier hospitals with abnormally high rates of observation (defined as those with rates of observation stay use at least 1.5 times the interquartile range for the national sample). Among the remaining hospitals, we excluded individual hospitalizations for hip and knee replacement (which were added to the HRRP later), a primary diagnosis including psychiatric conditions, rehabilitation, or cancers, and cases in which the beneficiary transferred to another facility, left the hospital against medical advice, or died in the hospital (*N* = 2,809,281). Finally, given evidence that CMS coding changes during HRRP implementation (which increased the number of diagnoses fields on a claims record from 9 to 25)^9^ may overestimate the HRRP effect, we limited capture of comorbidities to the first 9 diagnosis codes.

We identified hospitalizations for HRRP target conditions using clinical classification software (CCS) codes.^10^ We combined the first 3 conditions initially targeted by the HRRP (acute myocardial infarction, pneumonia, heart failure) into a single group for analysis. We analyzed COPD separately as it was added to the program later. To select an appropriate control group from conditions not targeted by the HRRP, we examined observation stays (defined by the presence of revenue center code 0760 or 0762) as a proportion of total hospitalizations for each condition in the pre‐period. Hospitalizations were classified as observation stays or inpatient admissions based on their status at discharge (i.e., observation stays that converted to inpatient admissions were classified as inpatient admissions). We observed substantial heterogeneity in this measure across conditions ranging from near 0% to over 50% of all hospital events. HRRP target conditions are predominantly managed via inpatient admission and had relatively low rates of observation use. Conditions with the highest rates of observation tended to be symptom diagnoses (e.g., chest pain, dizziness, syncope), which are qualitatively different from the targeted conditions. Thus, we elected to use a control group consisting of nontargeted conditions with observation stays comprising less than 10% of total hospitalizations in the pre‐period as our primary approach. This allowed us to compare changes in target and nontarget conditions primarily managed as inpatient admissions. However, we recognize this threshold is arbitrary, and conducted a sensitivity analysis using an alternative control group of all nontargeted conditions with parallel pre‐period trends.

For our primary analysis, we compared changes in the proportion of total hospitalizations billed as observation stays for the first 3 HRRP target conditions (heart failure, pneumonia, and acute myocardial infarction) and nontarget group over 3 periods: a 15‐month baseline period (January 2009–March 2010) preceding passage of the Affordable Care Act (ACA) and HRRP announcement, an intervening period (April 2010–September 2012) after the HRRP announcement but prior to HRRP penalty implementation, and a two‐year post‐HRRP penalty period (October 2012–September 2014) after penalty implementation. CMS announced that COPD would be added to the HRRP in August 2013 and instituted penalties beginning in October 2014. Thus, for the COPD analysis, we again used a 15‐month baseline period before the program announcement (May 2012–July 2013), an intervening period after the program announcement but prior to penalty implementation (August 2013–September 2014), and a 2‐year post‐HRRP penalty period (October 2014–September 2016).

As shown in equation 1, we estimated linear probability models within a DID framework to examine if the HRRP increased observation stay use among targeted conditions:

(1) $${\mathrm{Obs}}_{\mathrm{ijt}}=\alpha_{i}+\beta_{1}{\text{Target}}_{j}+\beta_{2}{\text{Month}}_{t}+\beta_{3}\text{Post}\_{\mathrm{ACA}}_{t}+\beta_{4}\text{Post}\_{\text{HRRP}}_{t}+\beta_{5}{\text{Target}}_{j}*{\text{Month}}_{t}+\beta_{6}{\text{Target}}_{j}*\mathrm{Pos}{t_{\mathrm{ACA}}}_{t}+\beta_{7}{\text{Target}}_{j}*\mathrm{Pos}{t_{\text{HRRP}}}_{t}+\beta_{8}{\text{Target}}_{j}*{\text{Month}}_{t}*\mathrm{Pos}{t_{\mathrm{ACA}}}_{t}+\beta_{9}{\text{Target}}_{j}*{\text{Month}}_{t}*\mathrm{Pos}{t_{\text{HRRP}}}_{t}+\beta_{8}{\text{Hospital}}_{j}+\beta_{9}X_{i}+\varepsilon_{i}$$

where ${\mathrm{Obs}}_{\mathrm{ijt}}$ is a binary indicator equal to 1 if a hospitalization for individual *i* with condition *j* at chronological month *t* was an observation stay and 0 otherwise. ${\text{Target}}_{j}$ is a binary indicator equal to 1 for HRRP target conditions and 0 for nontarget conditions, ${\text{Month}}_{t}$ is a continuous variable corresponding to calendar month, $\text{Post}\_{\mathrm{ACA}}_{t}$ and $\text{Post}\_{\text{HRRP}}_{t}$ are binary indicators denoting the corresponding policy periods, and $X_{i}$ is a vector of beneficiary characteristics including age, sex, race/ethnicity, comorbid conditions from the prior year, and CCS diagnosis. *Hospital* indicates the inclusion of hospital‐fixed effects. The interaction terms allow us to estimate changes in the rate of observation stay use over time for target versus nontarget conditions.

We also conducted several exploratory analyses. First, certain hospitals have higher readmission rates than others and are more likely to be penalized under the HRRP.^11^ CMS began reporting readmission rates in 2010 before the implementation of penalties. Thus, poor‐performing hospitals may have had incentive to change observation practices in anticipation of receiving penalties. Therefore, to examine whether hospitals penalized under the HRRP behaved differently with respect to their observation stay use, we stratified hospitals by their penalty status in 2013 (the first year that penalties were levied). Second, we categorized hospitals into quartiles based on the proportion of their hospitalizations that were observation stays (quartile 1 = lowest proportion to quartile 4 = highest proportion) and explored whether hospitals' baseline propensity to use observation stays might influence their use of observation stays in response to the HRRP. Third, we tested the use of 2 (rather than 3) policy periods, focusing on the period before and after HRRP announcement, and found similar results. We also estimated models controlling for the implementation of the Two‐Midnight Rule in October 2013 as a potential policy of interest but found no significant inflection points in observation stay use around that date. We conducted all analyses using Stata version 16.

## 3 RESULTS

**FIGURE 1:**
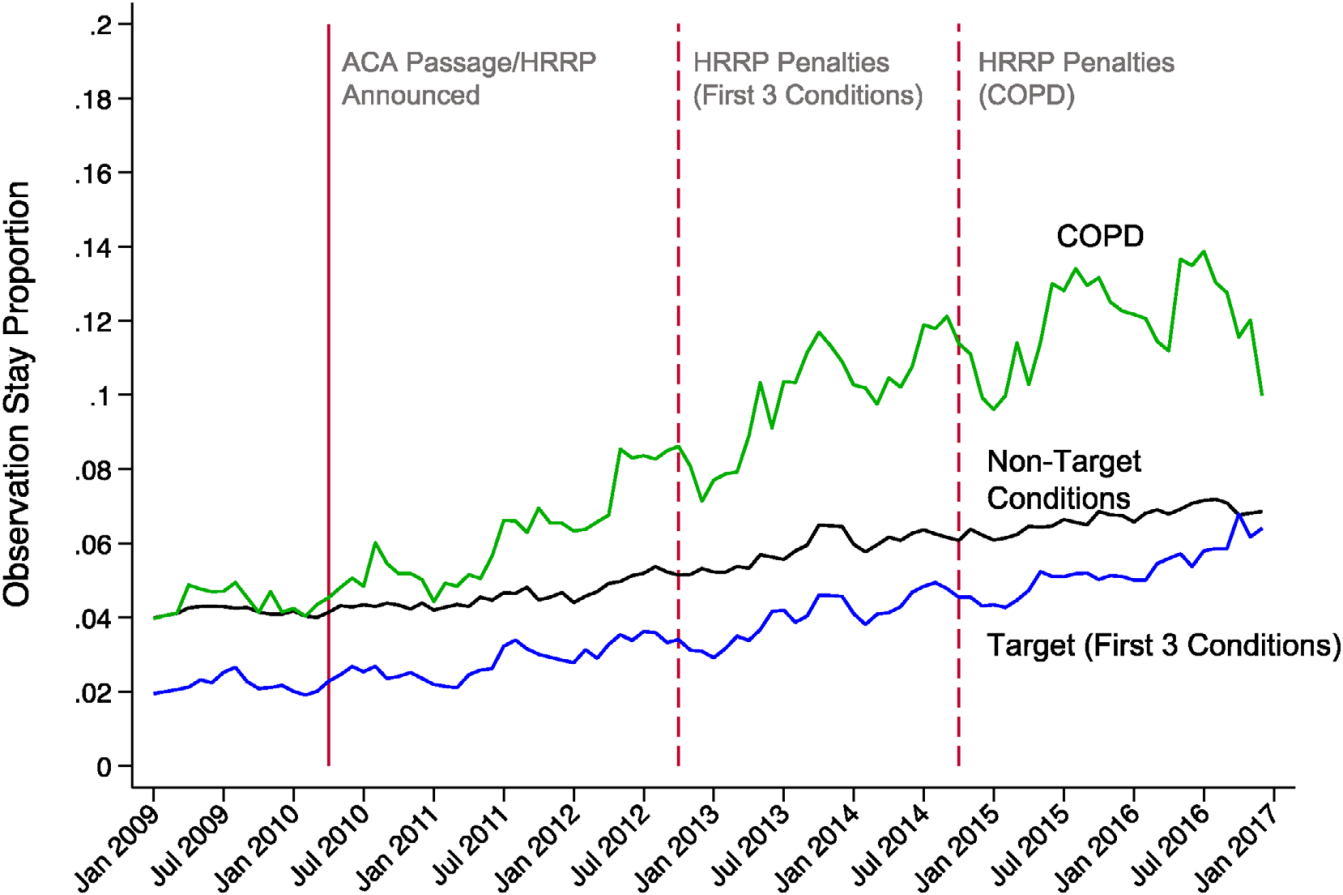
Unadjusted trends in observation stay use for target and nontarget conditions, 2009–2017. Target conditions include acute myocardial infarction, pneumonia, and heart failure. [Color figure can be viewed at wileyonlinelibrary.com]

Our primary analysis included 7,162,189 hospitalizations, of which 18.4% were for one of the first 3 HRRP target conditions. Unadjusted trends in observation stay use among our two target condition groups and our nontarget control group are shown in Figure 1. Table 1 shows the mean adjusted proportion of hospitalizations that were observation stays for the 3 policy periods (baseline, post‐announcement/pre‐penalty, post‐HRRP). Observation stays grew as a proportion of total hospitalizations throughout the study period for all conditions. For the combined target condition group, the proportion of hospitalizations that were observation stays remained relatively stable through the baseline period comprising about 2.2% of target hospitalizations and 4.2% of nontarget hospitalizations. In the post‐announcement/pre‐penalty period, observation stay use averaged 2.8% of target hospitalizations and 4.6% of nontarget hospitalizations. Finally, in the post‐HRRP period, observation stay use averaged 4.0% of target hospitalizations and 5.8% of nontarget hospitalizations.

**TABLE 1:** Adjusted mean observation stay use as a proportion of all hospitalizations.

Group	Baseline	Post‐announcement/ Pre‐Penalty	Post‐HRRP
Analysis of first 3 target conditions			
Combined target group	2.15	2.79	3.95
Acute myocardial infarction	0.11	0.91	2.20
Heart failure	3.67	4.30	5.46
Pneumonia	1.23	1.91	3.01
Non‐target group	4.16	4.57	5.83
Analysis of COPD			
COPD	8.41	7.56	11.85
Non‐target group	5.31	6.16	6.63

*Note*: Non‐Target conditions with observation stay proportion ≤10% included in control group. Policy periods vary based on the announcement and implementation of the HRRP for the condition group. For the combined target condition group: Baseline (January 2009–March 2010), post‐announcement period (April 2010–September 2012), and post‐HRRP period (October 2012–September 2014). Policy periods for the COPD group: Baseline (May 2012–July 2013), post‐announcement period (August 2013–September 2014), and post‐HRRP period (October 2014–September 2016).

Abbreviations: ACA, affordable care act; HRRP, hospital readmissions reduction program.

Our COPD analysis included 3,199,348 hospitalizations, of which 6.5% were for COPD. Observation stays initially comprised 8.4% of COPD hospitalizations and 5.3% of nontarget hospitalizations. After CMS announced the addition of COPD to the HRRP, observation stays averaged 7.6% of COPD hospitalizations and 6.2% of nontarget hospitalizations. In the post‐HRRP period, observation stays averaged 11.9% of COPD hospitalizations and 6.6% of nontarget hospitalizations.

The results of our fully adjusted DID models appear in Table 2. Relative to baseline, hospitals' observation stay use increased similarly in both target and nontarget groups (0.04 vs. 0.03% point increase per month) in the postannouncement period. This corresponds to an insignificant DID estimate of 0.01% points per month [95% CI −0.01, 0.3]. Observation stay use increased at a slightly higher rate among the target versus nontarget group in the post‐HRRP period (0.08 vs. 0.05% point increase per month) corresponding to a small but statistically significant DID estimate of 0.02% points per month [95% CI 0.002, 0.04].

**TABLE 2:** Estimated effects of the HRRP on hospital observation use.

	Baseline	Post‐ACA	Within group change	DiD	Post‐HRRP	Within group change	DiD
Analysis of first 3 target conditions							
Target	0.00068	**0.04475**	**0.04407**		**0.07638**	**0.07570**	
Non‐target	0.00586	**0.03960**	**0.03374**		**0.06043**	**0.05457**	
				0.01033			**0.02113**
Analysis of COPD							
COPD	**0.09382**	0.04677	−0.04705		**0.14091**	0.04710	
Non‐Target	**0.05068**	**0.01598**	**−0.03469**		**0.06151**	0.01084	
				−0.01236			0.03626

*Note*: Values reported are percentage point changes per month within each period. Bolded values indicate significant associations at the 0.05 alpha level. Difference‐in‐difference (DiD) effect modeled as interaction between linear month, policy period, and group. Policy periods vary based on the announcement and implementation of the HRRP for the condition group. For the combined target condition group: Baseline (January 2009–March 2010), post‐announcement period (April 2010–September 2012), and post‐HRRP period (October 2012–September 2014). Policy periods for the COPD group: Baseline (May 2012–July 2013), post‐announcement period (August 2013–September 2014), and post‐HRRP period (October 2014–September 2016).

Abbreviations: ACA, affordable care act; HRRP, hospital readmissions reduction program.

For the COPD analysis, hospitals' observation stay use was increasing at a rate of 0.09% points per month for COPD and 0.05% points per month for nontarget conditions. After the announcement that COPD would be added to the HRRP, observation stay use slowed for both the COPD and nontarget group (0.05 vs. 0.02% point increase per month), corresponding to an insignificant DID estimate of −0.01% points per month [95% CI −0.10, 0.08]. Finally, observation stay use increased at a higher rate for COPD compared to the nontarget conditions in the post‐HRRP penalty period (0.14 vs. 0.06% point increase per month), however, this corresponds to an insignificant DID estimate of 0.04% points per month [95% CI −0.03, 010]. In our sensitivity analyses, we did not identify any significant relationship between the HRRP and observation stay use for targeted conditions as a function of hospital penalty status or baseline propensity to use observation stays.

## 4 DISCUSSION

In this analysis, we found that observation stays increased over time at a similar rate for both HRRP target conditions and nontarget conditions. The only statistically significant DID estimate was for the original 3 HRRP target conditions in the post‐HRRP period, but even for this result, the effect size was quite small and its clinical significance is questionable. Our results are inconsistent with hospitals responding to the HRRP by managing patients through observation rather than inpatient admission and suggest that even if the HRRP did incentivize some additional observation stay use at the margins, it is not a major driver of the increase in observation stays. However, there are other potential explanations for our findings.

First, observation stays could increase across both target and nontarget groups if hospitals implemented broader policies and practices (e.g., placing more patients in observation) to reduce readmissions across all conditions. However, despite some evidence that the HRRP decreased readmissions for both target and nontarget conditions, the decrease was noticeably smaller among nontarget conditions—a finding consistent with hospitals focusing practices on target conditions.^12^

Second, our findings are likely influenced by Medicare's use of Recovery Audit Contractors (RACs) and Medicare Administrative Contractors (MACs) to conduct audits and ensure appropriate provider payments. The RAC program began as a demonstration project but was rolled out nationwide in fee‐for‐service Medicare in 2009 and Medicare Advantage in 2010 under the Tax Relief and Health Care Act of 2006.^13^ Thus, expansion of this program aligns closely with our post‐ACA period. Evidence suggests that RACs and MACs have focused on identifying short inpatient admissions that should have been billed as outpatient observation stays and hospitals have increased their use of observation stays to avoid audits.^14^ Indeed, RAC audits identified nearly $3.65 billion in overpayments in FY 2013,^15^ while HRRP penalties in FY 2022 were $521 million,^16^ which suggests that hospitals had more to gain by avoiding RAC audits than avoiding readmission penalties.^16^, ^17^ This would also explain the increase in observation stays across both target and nontarget conditions, since^13^ RAC and MAC audit activity is not condition‐specific.

Third, Medicare inpatient hospitalizations have gradually declined for the past few decades.^18^, ^19^ Thus, our findings may reflect general trends in clinical care delivery driven by a mixture of improvements in technology, patient preference, and physician risk aversion. For example, expansion of treatment in hospital outpatient departments may increase the ability of hospitals to care for less severely ill patients in alternative settings.^19^ In addition, with advances in chronic disease management and prevention, Medicare beneficiaries are living longer before progressing to severe disease, and this improvement in population health decreases their need for inpatient care.^20^ Simultaneously, the composition of fee‐for‐service Medicare beneficiaries has changed over time, with increased enrollment in Medicare Advantage plans, which may influence our results in unknown ways. However, examining these potential mechanisms is beyond the scope of the current study.

Overall, it does not appear that the HRRP led to meaningful changes in practice patterns that differentially affected the use of observation stays for target conditions. While this has remained an open question for a decade, it is finally time to put it to rest. Reclassifying a hospitalization from inpatient to observation can reduce reimbursement by more than 75%.^17^ By comparison, the average HRRP penalty was $217,000 (an 0.64% reduction in Medicare patient revenue) in 2018.^16^ Put simply, hospitals would forego far more in inpatient reimbursement than they would preserve by systematically using observation stays to avoid HRRP penalties. Our findings are likely due to other factors, including nationwide audit activity and clinical and technological advances.

While the HRRP does not appear to have induced hospitals to use observation stays, ignoring observation stays in readmission measures is likely to have other important implications for assessing program effects on hospital quality. Recent evidence suggests that including observation stays and other acute events like ED visits substantially changes the assessment of hospital quality.^21^ Failing to account for observation stays in the shifting landscape of hospitalization may make programs such as the HRRP less representative of hospital quality and use patterns over time and may misrepresent true performance based on local observation practices.^1^
